# Correlation between the histopathology of chronic urticaria and its clinical picture*

**DOI:** 10.1590/abd1806-4841.20165066

**Published:** 2016

**Authors:** Raquel Zappa Silva Marques, Roberta Fachini Jardim Criado, Carlos D'Apparecida Santos Machado Filho, Juliana Milhomem Tamanini, Cristina van Blarcum de Graaff Mello, Carolina Speyer

**Affiliations:** 1 Faculdade de Medicina do ABC (FMABC) – Santo André (SP), Brazil

**Keywords:** Biomarkers, Chronic disease, Urticaria

## Abstract

**BACKGROUND:**

Chronic urticaria is characterized by transient, pruritic lesions of varying sizes, with central pallor and well-defined edges, with disease duration longer than six weeks. Its cellular infiltrate consists of neutrophils, lymphocytes and eosinophils. There is a subgroup of patients with eosinophilic or neutrophilic urticaria, resistant to the treatment with antihistamines, but that respond to a combination of antihistamine with other drugs.

**OBJECTIVE:**

To evaluate the present infiltration in chronic urticaria biopsies and correlate it with the clinical disease activity and response to treatment.

**METHODS:**

Forty-one patients with chronic urticaria were classified according to the score of severity of the disease, response to treatment and type of perivascular infiltrate. Inflammatory infiltrates were divided in eosinophilic (46.30%), neutrophilic and mixed.

**RESULTS:**

An association was found between the eosinophilic infiltrate and clinical scores of greater severity (p = 0.002).

**CONCLUSION:**

This association shows that the eosinophilic inflammatory infiltrates denote high clinical activity, which means more severe and exuberant clinical pictures of the disease.

## INTRODUCTION

Urticaria is one of the most commonly conditions observed in dermatology daily practice, affecting 15% to 30% of the population.^1^ Wheal is its elementary dermatological lesion, characterized by being itchy and have central edema with varied size, surrounded by a reflex erythema and with ephemeral nature; furthermore, the skin returns to its normal aspect usually in a period that varies from one to 24 hours.^2^ Urticaria is classified, from the point of view of duration of its evolution, in acute (less than six weeks) or chronic (over six weeks).^3^

The physiopathology of chronic urticaria (CU) has long been associated with anxiety and allergy to foods or its additives, but today it is considered the participation of autoimmune mechanisms and coagulation factors.^4^ It is known that degranulation of mast cells or skin basophils is its initial stimulation. By releasing potent vasoactive mediators, vasodilation is induced, increasing capillary permeability and resulting in erythema and papule formation.^5^

The main vasoactive mediator involved in CU is histamine, however there are other mediators such as arachidonic acid metabolites, leukotrienes (LTC4, D4 and E4), prostaglandin D2, serotonin, acetylcholine, platelet-activating factor, heparin, codeine, anaphylatoxins C3 and C5a, cytokines and neurotransmitters released from cutaneous nerve endings.^6^ These mediators participate in attracting other inflammatory cells such as neutrophils, lymphocytes, and particularly eosinophils, which play a role in inflammation maintenance for release of other substances, characterizing the cellular infiltrate of the disease.^5^

Inflammatory infiltrates composed of neutrophils, eosinophils and their products - macrophages and helper T lymphocytes - have been described in cholinergic CU lesions and pressure urticaria. Increased vascular markers, eosinophils and neutrophils is a feature of damaged skin in CU, contributing to tissue edema.^6,7^ Also the increased Th2 cytokine expression in CU lesions suggests that the innate pathways may operate in the formation of these lesions by participating in mast cell activation, inflammation and the formation of vascular leakage.^8^

In addition, patients with CU often show signs of thrombin generation, as a result of the extrinsic pathway activation of coagulation and fibrinolysis, and have mean levels of D-dimer slightly higher, being a participant substance of this pathway.^4^

The clinical importance of characterizing the inflammatory infiltrate resides in the presence of a subgroup of patients presenting eosinophil or neutrophil urticaria, which may be resistant to treatment with antihistamines, but which responds to a combination of antihistamine with other drugs.^6^ In addition to the improvement and optimization of the treatment of disease, to establish a relation between inflammatory infiltrate and clinical score of severity of the disease can also aid in the prognosis of the disease and therefore in its management.

The aim of this study is to evaluate the inflammatory infiltrate present in CU biopsies and correlate it with the clinical activity of the disease and its response to treatment.

## METHODS

This is a cross-sectional, descriptive study of clinical basis. We selected 273 patients from the urticaria clinic of the Department of Dermatology of the Faculdade de Medicina do ABC, who were on routine outpatient treatment from January 2009 to May 2014. We excluded 229 patients without biopsy and three with biopsy compatible with urticarial vasculitis.

Our final sample consisted of 41 patients who underwent biopsy at some point in their treatment. The medical records of these patients were analyzed by a standard form containing the urticaria activity score (UAS) ^1^ and the biopsy report. The survey data was carried out by signing an informed consent by the patient during his/her routine consultations in the urticaria clinic.

To meet the inclusion criteria, patients had to: 1) come from the urticaria clinic; 2) present or have presented the disease for a period longer than six weeks; 3) be older than 18 years; 4) understand and sign the informed consent form; and 5) have undergone biopsy at some time of the disease.

Exclusion criteria were: 1) duration of urticaria shorter than six weeks; 2) failure to sign the informed consent form; 3) absence of biopsy of the lesions; and 4) age younger than 18 years. Patients with CU were classified according to UAS, which is determined by the number of daily urticaria and the intensity of pruritus,^1^ as shown in table 1. Clinical score is considered mild when UAS is from 0 to 2 points; moderate, when it is from 3 or 4 points; and high, when it is from 5 to 6 points.

**Table 1:** Urticaria intensity assessment

SCORE	WHEAL	ITCH
0	None	None
1	Mild (less than 20 wheals in 24 hours)	Mild
2	Moderate (21 to 50 wheals in 24 hours)	Moderate
3	High (more than 50 wheals in 24 hours or	Intense
	large confluent areas of wheals)	

Sum of scores (wheal + itch) = (0-6) Adapted from Darlenski *et al*^1^

The biopsy material was fixed in formalin 10% and stained with hematoxylin and eosin (HE) and subjected to microscopy at magnification of 400 times. The degree of perivascular infiltrate was considered as neutrophilic when the number of neutrophils was greater than or equal to seven out of 10 cells of the infiltrate; eosinophilic, when the number of eosinophils was greater than or equal to seven out of 10 cells of the infiltrate; and mixed, when there was no predominance of cells among monocytes, neutrophils and eosinophils.

CU treatment was performed with antihistamines and therapeutic monitoring, aiming to observe the degree of difficulty in controlling the disease. For patients with poor response (use of four or more doses of antihistmine per day) adjuvant treatment was used (cyclosporin, dapsone, montelukast, colchicine, chloroquine and corticosteroids).

Response to treatment was determined according to the number of daily doses of drugs in the control of urticaria. Response score was thus established: a dose of antihistamines - good answer; two or three doses - moderate response; and four or more doses - poor response. The criteria used for this control was the absence of urticaria or angioedema for 30 days, under the established treatment.

### Statistical analysis

The variable type of inflammatory infiltrate, PCR, D-dimer, clinical activity score and therapeutic response, for being categorical, were expressed in absolute and relative frequencies. Since the data were not normally distributed (Shapiro-Wilk test, p<0.05), IgE was expressed as medians and confidence intervals. To analyze the association between types of inflammatory infiltrates, inflammatory activity outcomes and response to therapy, we used the chi-square test. To analyze differences in IgE concentrations between the types of inflammatory infiltrates, we used the Kruskal-Wallis test. The significance level adopted was 95%. The software used was Stata 11.0.

## RESULTS

We selected 41 adult patients with biopsy at some time of treatment, 85.4% woman and 14.6% man. Inflammatory infiltrates were divided between eosinophilic, neutrophilic and mixed, and 46.30% represented the eosinophilic inflammatory infiltrate.

The type of inflammatory infiltrate observed in biopsies had no association with the value of IgE (Shapiro-Wilk test), but eosinophilic infiltrate was the one that presented higher median (value of 286). There was also no statistical significance between the D-dimer values and PCR and the type of inflammatory infiltrate (p = 0.709 and p = 0.508 respectively).

An association between eosinophilic infiltrate and clinical scores of greater severity (p = 0.002) was observed, as can be seen in table 2. There was no statistically significant association between the type of infiltrate and the therapeutic response (p = 0.535).

**Table 2:** Correlation between type of infiltrate and inflammatory activity tests, clinical score and therapeutic response

Activity		Inflammatory infiltrate — n (%)			p*
nflammatory activity		Eosinophilic	Neutrophilic	Mixed	
**CRP**					0.508
Less than 6		12 (66.7)	6 (60)	5 (45.5)	
Higher than 6		6 (33.3)	4 (40)	6 (54.5)	
**D-Dimer**					0.709
Less than 0.5		2 (22.7)	1 (12.5)	2 (28.6)	
Higher than 0.5		10 (83.3)	7 (87.5)	5 (71.4)	
**Clinical activity**					0.002
Mild		2 (10.5)	0	4 (36.3)	
Moderate		2 (10.5)	6 (75)	4 (36.3)	
High		15 (79)	2 (25)	3 (27.4)	
**Therapeutic response**					0.535
Good response		5 (26.32)	1 (10)	2 (28.57)	
Moderate response		7 (36.84)	5 (50)	5 (57.14)	
Poor response		7 (36.84)	4 (40)	1 (14.29)	

* Probabilistic value of chi-square test with significance level of 95%; CRP: C-reactive protein

## DISCUSSION

Our study showed a higher percentage of female patients, which is in accordance with the demographic profile of the disease that is already known, but the proportion found was 5.84 women for one man, while in literature the proportion is 2:1.^9^ The type and intensity of the inflammatory infiltrate seen by biopsy of the lesions were quite variable and depended on the time when the biopsy was performed and on the etiology of the lesions. It is common the presence of edema in the reticular dermis, and the cellular infiltrate may have a predominance of neutrophils, eosinophils or both, featuring its histological type.^10^

In our study, an association between the inflammatory infiltrate and the clinical score of the disease was demonstrated: eosinophilic type showed high clinical activity, i.e., more serious and exuberant clinical pictures of the disease, while neutrophilic and mixed infiltrate types manifested clinically in a mildest form.^11^ The tissue factor expressed by eosinophils induces the activation of blood clotting and thrombin generation, which, in turn, can increase vascular permeability, both directly, acting on endothelial cells, and indirectly, inducing mast cell degranulation by the release of histamine.^12^ Thus, the association found in this study between the clinical score of greater severity and eosinophil infiltrate shows that, when the eosinophil predominates, there is more than an activation pathway of the disease - the direct and also the indirect – reinforcing its development and aggravating its clinical picture.

In our study, the relation between the type of infiltrate and response to treatment wasn't identified. Urticaria presents a wide range of etiologies, and its onset comes from different cellular activation orders. Thus, the therapeutic response is very variable and susceptible to idiosyncrasies, because each patient has a trigger mechanism of the disease.

In our study, no statistical significance between the values of IgE and the types of inflammatory infiltrate was found, but the median IgE in eosinophilic infiltrate presented higher than in the other infiltrates. IgE is produced by plasma cells, has a short mean life and receptors in various inflammatory cells, among them mast cells and basophils, which have specific Fc receptors.^11^ Mast cells are potent inflammatory cells that express on their surface receptors able to start, expand and perpetuate inflammatory processes by releasing soluble factors and that interact with other immune effector cells.^11^

In our study, no relation between D-dimer and inflammatory infiltrate was evidenced, although there is in the literature a relation between disease severity and high plasma levels of D-dimer.^4^ Also, relation between values of CRP and infiltrate was not identified, but this is a unspecific marker of systemic inflammation.^13^

## CONCLUSION

In our study, we found significant statistical relation between the type of inflammatory infiltrate, characterized in histopathological analysis of biopsies, and clinical score of disease severity, according to Zuberbier criteria:^1^ eosinophilic infiltrates presented relation with more severe clinical manifestations of the disease, while neutrophilic infiltrates showed a relation with milder clinical pictures.

Relation between the findings of biopsies and response to treatment of urticaria was not demonstrated. Also, the study didn't find significant statistical relation between the infiltrate and CRP, D-dimer and IgE variables.
